# Supplementary material for: Sesamol Attenuates Renal Inflammation and Arrests Reactive-Oxygen-Species-Mediated IL-1β Secretion via the HO-1-Induced Inhibition of the IKKα/NFκB Pathway In Vivo and In Vitro
Source: Antioxidants (Basel). 2022 Dec 14;11(12):2461. doi: 10.3390/antiox11122461 (PMC9774643; doi:10.3390/antiox11122461)
Supplement: Supplementary file 1 [file antioxidants-11-02461-s001.zip › antioxidants-2042462-supplementary.pdf]

Residues interactions
1N3U GLN 212 ↔ 5EBZ GLY 525;
1N3U GLN 212 ↔ 5EBZ VAL 526;
1N3U GLN 212 ↔ 5EBZ GLY 528;
1N3U LEU 220 ↔ 5EBZ ILE 519;
1N3U THR 205 ↔ 5EBZ ASP 532;
1N3U ARG 198 ↔ 5EBZ SER 536;
1N3U ALA 194 ↔ 5EBZ GLU 540;
1N3U ALA 194 ↔ 5EBZ GLU 543;
1N3U ALA 194 ↔ 5EBZ LEU 544;
1N3U GLU 120 ↔ 5EBZ GLN 533;
1N3U GLU 219 ↔ 5EBZ ALA 522;
1N3U HIS 223 ↔ 5EBZ HIS 484;
1N3U LEU 208 ↔ 5EBZ ASP 532;
1N3U GLN 197 ↔ 5EBZ GLU 543;
1N3U ARG 117 ↔ 5EBZ ASP 532;
1N3U GLN 212 ↔ 5EBZ TYR 529;
1N3U HIS 223 ↔ 5EBZ ILE 519;
1N3U GLU 201 ↔ 5EBZ SER 536;
1N3U HIS 223 ↔ 5EBZ GLU 515;
1N3U GLU 201 ↔ 5EBZ ALA 539;
1N3U PRO 193 ↔ 5EBZ GLU 543;
1N3U ARG 113 ↔ 5EBZ VAL 526;
1N3U ARG 198 ↔ 5EBZ GLU 540;
1N3U GLU 219 ↔ 5EBZ ILE 519;
1N3U ARG 113 ↔ 5EBZ TYR 529;

Supplementary Figure S1. Residues interaction for the interaction of IKKα (PDB code: 5EBZ; red structure) and HO-1 (PDB code: 1N3U; blue structure).
